# Fractures Treated at the Buffalo General Hospital

**Published:** 1872-01

**Authors:** C. C. F. Gay


ART. II.—*Fractures Treated at the Buffalo General Hospital. By* C. C. F. GAY, M. D.

During my term of service for 1870 and 1871 there were treated twenty-six fractures. For purposes of advancing surgical science of fractured bones, the individual fractures named in the following table cannot be given with sufficient accuracy and minuteness. For instance, in some of them the precise point of lesion and whether the fracture was oblique or transverse, are not stated, neither is the cause given in the hospital records. The records are deficient in not stating also the manner in which the injury was inflicted. More attention will hereafter be given to make the records more complete, so that the cases may be reported in a way that will, it is hoped, contribute something to the science and art of this branch of the profession.

With these remarks I will give the table of fractures that have been furnished me by the House Physician, Dr. Harrington ;

I will premise, however, by saying that these fractures are those only treated during the writer's term of service, having no reference to those treated in the same institution during the service of my colleagues.

Scapula corocoid, process of. Ribs, 11th. do. 10th. Clavicle, right side. do. both. Humerus, upper third, oblique. do. Fore-arm, radius and ulnar. Ulnar and radius. Ulnar Ulnar. Radius. Femur, neck of. do. do. Tibia, (internal maclulus). do. do. do. refracture of. do. compound comminuted. Fibula. do. Miscellaneous fractures. Skull, death occurred in forty-eight hours. Leg and ankle, compound comminuted, amputated. Leg, both, compound comminuted, death occurring in six hours. Leg and arm, compound; died in twenty-four hours. Thigh, compound comminuted, amputation; died in twenty-four hours.

Of the two cases of fracture of the neck of the thigh, one remained in hospital seven weeks, the other eight weeks.

The result in one case is not known; there was shortening in the other of two and a half inches. This patient had been under treatment for three weeks prior to his coming to the hospital. Both extension and counter-extension were employed. The ages of these two patients respectively were 34 and 64 years. Thomas, aged 34 years, whose leg was shortened two and half inches, received his injury from a direct blow. He was somewhat broken down in health by previous rheumatism.

One case of fractured clavicle was treated by the method recently advised by Dr. Moore, of Rochester, but I am sorry to say with no better result than the others treated by adhesive straps alone.

Two cases of fracture of internal malleolus were treated without splints, there was no displacement; therefore refrigerating lotions, alternating with fomentations, were employed for a few days until inflammation was subdued, when a simple roller was applied around the foot and leg. In one case a bad form of erysipelas supervened, which terminated in recovery. In the other case the result was perfect.

The simpliest appliances were used in all the other fractures, both of the arm, fore-arm and leg, and the apparatus removed in from fifteen to thirty days, the result being perfect in all the cases, save those known on their entrance to hospital to be so severe and complicated that deformity was inevitable.

One case of compound comminuted fracture of both legs terminated fatally in six hours from shock of injury.

One case of comminuted fracture of tibia, necessitated removal of a portion of tibia three inches long. The patient recovered with a good result.

One of the fatal cases was complicated by a burn from a gas well. In a fit of delirium he got out of bed, fell over the banisters, breaking his thigh at its lower third. The upper fragment projected beyond the integrements three inches, which was sawn off by the chain saw, the fractured bones then were properly adjusted, but the patient only survived the double shock from burn and fracture a couple of days.

In a case of fractured fibula, as there was no displacement, no splints were made use of. The patient placed in bed for two weeks and ordered to keep quiet; result perfect.

An instance, showing the importance of rest and quietude, occurred in the person of one who was injured upon the railroad. He had but a single rib broken, one of the fractured extremities inclined greatly to protrude. A compress and adhesive bandage were used, and the patient seemed progressing as well as any one could with such injury. In three or four days he determined to get up and go home to Canada to his friends. He left under protest, never obtaining the assent of either the attending or house surgeon to leave, but self-willed as he was, he left and died before he reached his friends in Canada, probably within forty-eight hours after leaving the hospital.

I regret that dextrine, so much used in the Massachusetts General Hospital for the treatment of fractures, has never been introduced here. From what I know of it I am convinced of its great superiority over many other forms of dressing.

